# Recent Mitigation Strategies on Membrane Fouling for Oily Wastewater Treatment

**DOI:** 10.3390/membranes12010026

**Published:** 2021-12-25

**Authors:** Nur Fatihah Zulkefli, Nur Hashimah Alias, Nur Shafiqah Jamaluddin, Norfadhilatuladha Abdullah, Shareena Fairuz Abdul Manaf, Nur Hidayati Othman, Fauziah Marpani, Muhammad Shafiq Mat-Shayuti, Tutuk Djoko Kusworo

**Affiliations:** 1 Department of Oil and Gas Engineering, School of Chemical Engineering, College of Engineering, Universiti Teknologi MARA, Shah Alam 40450, Malaysia; nurfatihahzulkefli@gmail.com (N.F.Z.); nurshafiqahjamaluddin96@gmail.com (N.S.J.); shareenafairuz@uitm.edu.my (S.F.A.M.); nurhidayati0955@uitm.edu.my (N.H.O.); fauziah176@uitm.edu.my (F.M.); mshafiq5779@uitm.edu.my (M.S.M.-S.); 2 Advanced Membrane Technology Research Centre (AMTEC), School of Chemical and Energy Engineering, Universiti Teknologi Malaysia (UTM), Johor Bahru 81310, Malaysia; fadhilatuladha@gmail.com; 3 Department of Chemical Engineering, Faculty of Engineering, Diponegoro University, Semarang 50275, Indonesia; tdkusworo@che.undip.ac.id

**Keywords:** fouling, membrane, oily, wastewater, mitigation, treatment

## Abstract

The discharge of massive amounts of oily wastewater has become one of the major concerns among the scientific community. Membrane filtration has been one of the most used methods of treating oily wastewater due to its stability, convenience handling, and durability. However, the continuous occurrence of membrane fouling aggravates the membrane’s performance efficiency. Membrane fouling can be defined as the accumulation of various materials in the pores or surface of the membrane that affect the permeate’s quantity and quality. Many aspects of fouling have been reviewed, but recent methods for fouling reduction in oily wastewater have not been explored and discussed sufficiently. This review highlights the mitigation strategies to reduce membrane fouling from oily wastewater. We first review the membrane technology principle for oily wastewater treatment, followed by a discussion on different fouling mechanisms of inorganic fouling, organic fouling, biological fouling, and colloidal fouling for better understanding and prevention of membrane fouling. Recent mitigation strategies to reduce fouling caused by oily wastewater treatment are also discussed.

## 1. Overview on Oily Wastewater

Industries such as food, petrochemicals, and petroleum refining generate oily wastewater that pollute soil and water and intoxicates the human body system [1]. Quantitatively, the world’s total volume of oily wastewater reached 10–15 billion m^3^ in 2013, and this figure is expected to grow dramatically over the years [2]. Generally, the generated oily wastewater is commonly characterised by the presence of salts, impurities and suspended oil droplets and greases [3]. Oily wastewater seems not to be a new concern in water contamination studies [4,5,6,7,8]. However, direct disposal of this wastewater is now restricted by state legislation, as it would result in severe water and soil contamination. The typical spectrum of oil compositions is between 100 to 1000 mg/L, with the allowable disposal boundaries of 10 mg/L for inland surface water and 20 mg/L for coastal marine areas, accordingly [9,10]. Oily wastewater is generally discharged from various sources, for example, car production facilities [11], machinery, metal production, offshore oil extraction, refining, oil and gas drilling [12], oil transport and oil distribution. As a result of oil usage from these various industries, a high amount of oil is dumped into rivers and water sources. This uncontrollable rise in the discharged volume of oily wastewater in different forms such as emulsion (droplets of oils are dispersed throughout the water), inverse emulsion (droplets of water dispersed within the droplets of oils), total dissolve solid (particles are not able to filter out through the filtered paper and settle to the bottom) and suspended solid (floating in the water rather than dispersed) can lead to environmental and surrounding issues [13]. Therefore, various research and development in technologies were evaluated for the treatment of oily wastewater until today. Over the last decades, one of the most popular treatments for oily wastewater has been skimming. Skimming is a simple process based on gravity separation. The oil can be removed by promoting a good density difference in which oil rises to the top of separator while the suspended solids sink downward [14]. The advantage of skimming is that the design system is straightforward, but the process is not suitable for treating emulsified oil since the oil droplets are small [15]. In addition, the skimming device also generates a high volume of sludge, resulting in additional treatment [15].

Conversely, dissolved air floatation (DAF) is a physical floatation method for oily wastewater, including emulsified oil with less sludge generation. Generally, air is introduced under pressure at the bottom of basin [16,17]. The bubbles generated from the DAF process range from 20 to 100 microns under atmospheric conditions [16]. As the bubbles rise from the bottom of the basin, the pollutants will attach to the bubbles. Several studies have been reported DAF capable of removing higher than 90% oily wastewater [18]. However, the main drawback for DAF is that the process requires a high capital cost. Besides DAF, coagulation coupled with flocculation (coagulation/flocculation) is a popular process to remove oil from wastewater. Coagulation/flocculation has a lower operational cost and is much easier to operate [2]. The most common principle of the process is that oil is removed as it floats on the water’s surface during the floatation process.

Consequently, coagulants or flocculants are added to the wastewater to destabilise the remaining suspended solids, oils particle, and colloids and develop flocs by neutralising the negative charge of oil emulsion [2]. Finally, the flocs are removed by sedimentation. However, the process generates a large volume of sludge that needs further treatment and increased operational costs [16]. In this regard, the adsorption process has been widely investigated for oily wastewater treatment because less or no sludge is produced at a low cost [19]. Various adsorbents treat oily wastewater such as agriculture waste, activated carbon and chitosan [19]. However, adsorption suffers from low separation efficiency [20]. Recently, the biological treatment also received considerable attention for oily wastewater treatment. Thus, a variety of microbes has been used for oily wastewater under different operating conditions. The treatment shows notable effectiveness in most of the studies. Although biological treatment is undoubtedly efficient, the development of biological treatment involves a complex procedure due to the diverse behaviours and nature of microorganisms under different environmental conditions. The process also generates a huge volume of sludge [4]. Table 1 summarises the common technology for the treatment of oily wastewater with its advantages and disadvantages.

In summary, most of these methods can efficiently treat oily wastewater. Still, they have several drawbacks, including generating secondary pollutants, having high maintenance costs, and being ineffective in separating emulsion [21,22]. As compared to these methods, membrane technology is one of the most effective among these processes, as it can be broadly used for the handling of oily wastewater due to advantages such as its high separation performance, more straightforward process, low energy consumption, incredibly compact model and limited space requirements [22,23]. With these superior advantages, membrane filtration has emerged as a promising alternative for oily wastewater treatment.

**Table 1:** Summary of common technology for the treatment of oily wastewater with their main advantages and disadvantages.

Method	Advantages	Disadvantages	The Extent of Oil Removal in Effluent Concentration	Reference
Skimming	- Simple process	- Unsuitable for emulsified oily wastewater and high sludge generation	N/A	[14,15]
Dissolve air floatation	- High removal efficiency	- Less efficient to separate oil droplet < 20 micron	95% removal	[16,17,18,24,25,26]
Coagulation /Flocculation	- Less sludge generation	- Requires high amount of coagulants	90% removal	[16,19,27]
Biological treatment	- High removal efficiency and environmentally friendly	- Time-consuming	98% removal	[4,25]
Adsorption	- Simple process with less sludge generation	- Low separation efficiency	67% removal	[19,20]

In brief, membrane filtration can be divided depending on the molecular weight cut off (MWCO), which are microfiltration (MF), ultrafiltration (UF), nanofiltration (NF) and reverse osmosis (RO) [26]. Commonly, the membrane can be fabricated by using either polymer-based organic membranes or inorganic ceramic membranes. The application of the precursor membrane materials depends on the water treatment process [27].

While membrane filtration is energy efficient, easy processing, and has low maintenance cost, membrane filtration suffers from membrane fouling [28]. Membrane fouling is a condition where membrane efficiency is jeopardised by a substance or matter on the surface and within membrane pores [29]. Membrane fouling not only causes flux decline but requires extreme costly chemical cleaning to reduce the impact of fouling. Oily wastewater membrane separation is essentially focused on two results: exclusion of the size and selective wettability [30]. The size exclusion indicates that the membrane allows water to move over the pressure exerted while inhibiting oil droplets larger than the membranes’ pores [31]. The selective wettability ensures that oil droplets do not penetrate the membrane’s pores by selecting water and oils properties such as hydrophilicity and oleophobicity underwater [32]. Membrane fouling has been widely studied to understand the mechanism and reduce fouling impact. However, this remains one of the critical problems of water sector membrane technology [28].

As many aspects of fouling have been reviewed, the current trend of methods of fouling reduction in oily wastewater have not yet been thoroughly discussed. Hence, this review is intended to discuss mitigation strategies to reduce membrane fouling from oily wastewater treatment. First, a general overview of membrane technology’s principal for oily wastewater treatment and fouling behaviour on the membrane will be briefly discussed, with subsequent further discussion on the current trend of methods used to mitigate the impact of fouling caused by treating oily wastewater.

## 2. Principal of Membrane Technologies for Oily Wastewater Treatment

Membrane technology has been applied for water/wastewater treatment since the 1960s. In general, the composition of membrane material can be mainly categorised into organic and inorganic, where organic membranes are usually composed of polymer. In contrast, inorganic membranes are made of ceramics or glasses [33]. Although membrane technology is useful for treating oily wastewater, membrane fouling is the biggest crisis, leading to a loss of productivity over time and requiring post-cleaning chemicals that contribute to operating and investment expense [34]. In addition, although the polymeric membrane is widely applied for water treatment, the hydrophobic nature of polymers interact well with oil and consequently cause membrane fouling [35]. Therefore, chemical cleaning is implemented on a routine basis to preserve membrane efficiency and reduce the fouling effect. However, continuous use of these acidic and alkaline chemicals negatively impacts people and the environment caused by the generation of secondary contaminants.

Moreover, it significantly reduces the membrane lifetime by causing membrane degradation [36], and thus various alternatives have been proposed. Therefore, before applying the advanced mitigation strategies for membrane fouling, it is suggested to understand the membrane technology principle that influences the membrane fouling effect of oily wastewater. Recent studies show that membrane fouling can be controlled by altering these two leading factors in the membrane process: (i) membrane properties and (ii) the effect of surfactants [37,38].

### 2.1. Membrane Properties to Treat Oily Wastewater

Membrane properties play a crucial role in controlling the fouling of the membrane. To control the membrane’s fouling, pore size distribution, surface roughness, and surface charges of the membrane are three major aspects that should be considered. Membrane with wide pore size distribution exhibit high fouling of oily wastewater due to pore-clogging. Conversely, narrower pore size distribution can help to minimise the fouling as it will reject the wider range of particles. A study revealed a higher fouling potential was seen at a membrane with a larger pore size (300 nm) than 80 nm pore size [39]. Therefore, from a practical point of view, the membrane pore size must be sufficiently narrow to prevent fouling for oily wastewater treatment.

In terms of membrane surface roughness, a membrane with a rougher surface or more hydrophobic is more susceptible to fouling because foulants can easily deposit on the membrane surface [40,41]. In addition, hydrophilic membranes tend to reduce the occurrence of fouling by providing greater surface bonding of a water layer while fabricating membranes with similar charges to contaminants [42,43]. Generally, the roughness of the membrane also depends on the porosity of the membrane. As the porosity of the membrane decreases, the surface roughness of the membrane also changes, thus increasing the transmembrane pressure (TMP) and the possibility of adsorbing contaminants on the membrane surface [44]. Thus, narrow pore size distribution with high porosity membrane is commonly preferred to treat oily wastewater. The effect of surface charge of membrane plays an important role in membrane fouling phenomenon. In general, membrane fouling is promoted by the electrostatic attraction between membrane and oil droplets. Many studies found that a membrane with a similar charge with an oil droplet can prevent fouling effectively [39]. Nonetheless, a previous study has successfully demonstrated that a zwitterionic membrane with surface chemistry is also excellent against fouling [45].

### 2.2. Effect of Surfactants

Surfactants are commonly present in membrane technology as an additive for oily wastewater treatment to produce well-stabilised oil emulsions. Surfactants minimise oil–water interfacial stress when the oil mixture is separated to the oil–water interface, thus reducing the energy needed for droplet breakup [46,47,48] Yet, the presence of surfactant will modify emulsion properties, including interfacial stress, droplet size and charge, and membrane properties, such as wetting and surface charge. Membrane properties such as surface charge [47,48] and water and oil hydrophilicity and oleophilicity [49,50] can be altered by surfactants. The surfactant’s ability varies based on the form and composition of both oil and the surfactant types, mixture conditions, temperature and phase composition [51,52]. For example, a membrane with hydrophilic properties may become more oleophilic and less hydrophilic upon the adsorption of surfactants. As the type of surfactant varies, such as cationic, anionic, and zwitterionic, the surface charge and membrane fouling tendency depend on the type and quantity of surfactant added. A study carried out by Xiabou et al. [53] reported that stabilised emulsion after adding anionic and non-ionic surfactants experienced less fouling but cationic surfactant easily fouled by negatively charged UF membrane. Usually, the change in surface charge of the membrane is generally characterised by the surface tension that controls the adsorption of surfactants and the adsorption mode via zeta potential analysis.

## 3. Fouling Behaviour on Membrane Filtration

As we are aware, membrane fouling is considered the main issue that decreases the membrane’s performance and restricts wider applications of the membrane. In general, fouling is defined as the membrane–solution interaction that causes accumulation of suspension or dissolved solids either on the surface of the outer membrane, on the membrane’s pores, or within the membrane’s pores [54]. Membrane fouling can be classified into four types: organic precipitation, colloids, inorganic precipitation, and biofoulings [55,56]. Colloids refer to the various particle size of colloids ranging in size from several nanometres to micrometres. Colloids can be categorised according to their size.

Furthermore, aquatic colloids can also be classified based on their dispersed compound, either organic or inorganic [56]. Organic colloids that have been frequently reported are fats, carbohydrates, proteins, greases, and surfactants are examples of organic colloids. In contrast, inorganic colloids include silica sediments, crystal and silt [57]. Regardless of their type and size, all colloids can cause colloidal fouling and impair membrane separation performance. Next, biofouling can be explained as the accumulation and adhesion of microorganisms [56,57]. Bacteria and fungi are highly reported microorganisms that account for the total membrane fouling [58]. Therefore, a membrane with a smooth surface with high hydrophilicity was suggested to reduce the chances of biofouling.

Conversely, organic fouling occurs from the accumulation of organic compounds. Several studies reported that the deposition of organic substances commonly found in the membrane separation process is from proteins, polysaccharides, nucleic acids, amino acids, and lipids. Lastly, inorganic fouling is generally from the deposition of inorganic compounds. The deposition could be either on the membrane surface or in between the membrane pores [59].

Therefore, physicochemical cleaning is required to remove the foulants effectively, but this approach increases operating costs, reduces membrane lifespan and durability and increases energy consumption. In this regard, a theoretical purpose of fouling control is to prolong and reduce fouling optimally and eliminate the accumulated foulants [60,61]. The following sections first elaborate the fouling mechanisms on membranes based on oil droplet behaviour on membrane and fouling models.

### 3.1. Fouling Mechanism on Membranes

#### 3.1.1. Wetting Behaviour of Oil Droplets on Membrane

**Figure 1:**
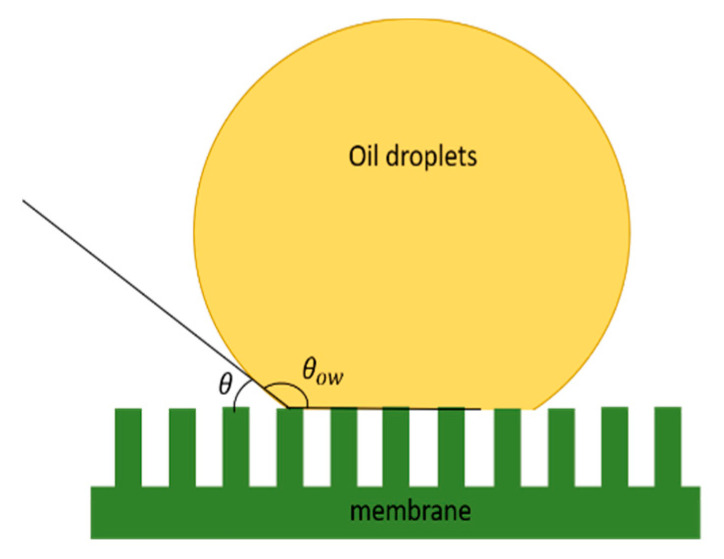
Illustration of an oil droplet attached on the surface of the membrane.

In oil–water separation, wetting behaviour on the membrane surface is crucial to determine the ability of one solid surface to absorb water molecules and repel other compounds. For this reason, researchers have developed switchable filter membranes with switchable wettability on textiles, carbon nanotubes fabrics, and filter paper to achieve smart oil and water separation [62]. Figure 1 shows the illustration of oil droplets attached to the surface of the membrane.

**Figure 2:**
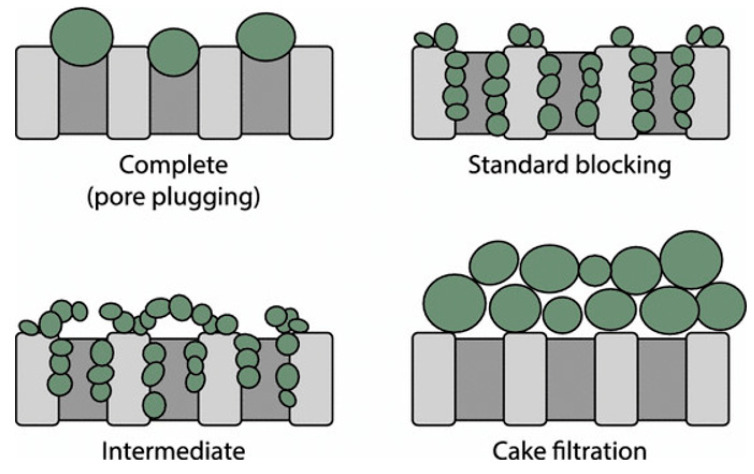
Illustration of several membrane fouling mechanisms [72].

In general, membrane surface wettability can be categorised into four regimes: (1) superhydrophobic (water contact angle > 150°), (2) hydrophobic (water contact angle > 90−150°); (3) hydrophilic (water contact angle < 90°) and (4) superhydrophilic (water contact angle ~0–10°). For the case of low surface tension liquid such as oil, the generalisation can be classified as (1) superoleophobic (oil contact angle > 150°); (2) oleophobic (oil contact angle > 90°); (3) oleophilic (oil contact angle < 90°); and (4) superoleophilic (oil contact angle~0–10°). The attachment of oil droplets to measure surface wettability is illustrated in Figure 2. Oil droplets can permeate the membrane at applied pressure greater than the critical pressure [1,63]. According to the reference article, the critical pressure can be calculated by using the following Equation:

(1) $$P_{crit}=2\gamma_{ow}\frac{\cos\theta }{r_{pore}}\left[1-\left\{\frac{2+3\cos\theta -cos^{3}\theta }{4{\left(\frac{r_{drop}}{r_{pore}}\right)}^{3}cos^{3}\theta -\left(2-3sin\theta +sin^{3}\theta \right)}\right\}\right]$$

where $\gamma_{ow}$ is the interfacial tension between oil and water, while θ indicates the contact angle from the water $r_{pore}$ and the $r_{drop}$ represent the radius of pores and the radius of oil droplets. Various assumptions can be made based on the Equation above, one of which is the contact angle will determine the sign of the critical pressure, whether negative or positive. First, the oil droplets can spontaneously penetrate through the membrane’s pores regardless of the pressure and lead to the oil’s failure to filtrate [64]. Next, when the contact angle is more than 90°, the critical pressure will increase and thus reduce pore radius, where we can assume that smaller pores contribute to higher oil droplet rejection and vice versa [1]. The wettability calculation is useful to assume the effect of wettability on the separation efficiency of oily wastewater and the fouling effect.

#### 3.1.2. Membrane Fouling Models of Oil

Many researchers have explored the membrane fouling models as they could provide an understanding of membrane fouling phenomena. Generally, there are four classic models: complete blocking, intermediate blocking, standard blocking, and cake layers [65]. Table 2 depicts the description of membrane fouling models. The complete blocking principle is based on pore trapping. It is presumed that each particle enters an empty pore inside the membrane and seals the pore opening entirely without overlaying on other particles. Therefore, a complete blocking model applies to membrane structures with smaller pores and in contact with larger contaminants.

Nevertheless, the number of pores that are being sealed increases correspondingly to the volume of the filtrate, while the diameter of the pores remains constant [66]. In general, membrane fouling can be classified into reversible and irreversible, as shown in Table 3. The reversible fouling resistance is commonly washed by physical means, such as backflush or changing the feed with fresh water, while the irreversible membrane fouling requires chemical cleaning [67]. Reversible and irreversible fouling usually develop instantly at the start of filtration; however, it slows at long-term processing.

**Table 2:** Phenomenal background and effect of mass transport of fouling mechanism during cross-flow filtration [55].

Fouling Mechanism	N	Background	Effect Mass Transport
Complete (pore plugging)	2	The oil droplets completely block the pore of the membrane since the size is larger.	The active site of the membrane decreases depending on the velocity of the feed
Internal pore-blocking/standard blocking	1.5	The oil droplets are either absorbed or deposited on the membrane walls since the size is smaller and restricts the flow of permeate.	Membrane resistance increases due to pore size reduction. Internal pore blocking is independent of feed velocity. Mitigation by cross-flow is absent.
Particle pore-blocking/intermediate	1	The oil droplets seal or bridge the pores or partially block the pores.	Reduction of active membrane area. The effect is similar to pore blocking but is not as severe.
Cake filtration	0	The oil droplets neither enter nor seal the pores, resulting in cake layer formation.	The overall resistance becomes the resistance of the cake plus the resistance of the membrane.

**Table 3:** The typical range of different fouling rates occurring at full scale [68].

Category	Fouling Rate (mbar/min)	Time Frame
Reversible fouling	0.1–1	10 min
Irreversible fouling	0.001–0.01	6–12 months

Additionally, fouling mechanisms are considered to occur simultaneously. The common manner of fouling always starts internally, followed by pore blockage and, lastly, cake formation on top of the membrane surface. During filtration of oily wastewater, emulsified oil droplets are in contact and deposited on the surface of the membrane [69]. At the early stage of filtration, the accumulated droplets will partially block the membrane pores. However, pore-blocking actions are fundamentally different from each other. The illustration of several membrane fouling mechanisms is depicted in Figure 2. Based on the figure, the deposition of oil droplets onto the membrane can be divided into internal and external fouling. Internal fouling occurs when oil droplets are deposited or absorbed inside the pores of the membrane. In contrast, external fouling occurs only on the surface and becomes a cake layer over time [70]. Pore blocking is one of the most commonly used terms to describe the flux decrease in membrane filtration.

Based on the intermediate blocking model, not every foulant particle is closely interacting with the pores, but a few sits on top of others. Large quantities of foulant particles aggregate on the membrane in the cake filtration model and form a cake layer which places greater resistance to the permeate flow. Such models predict various permeate flux decline patterns during filtration. They are used to evaluate experimental findings in the treatment of oily wastewater using membranes [71]. Combining these fouling models results in the entrance of foulants, and their accumulation on the surface of the membrane may lead to irreversible fouling. Membrane fouling is predicted to be more difficult for oily wastewater treatment since membrane surface and pores may be wetted with oil droplets, and the oil droplets can accumulate on the surface can transform during filtration and recrystallisation. These specific behaviours strongly impact the fouling of membranes during oily wastewater treatment [1].

The wettability of the surface accumulation and the physical membrane based on total filtration resistance have been reported by several studies. This method shows that attention should be given to the resistance in series model, where cumulative fouling could be described as the total of the various contributions associated with particular fouling mechanisms [73,74]. Equation (2), derived from Darcy’s rule, should convey the overall resistance to filtration [75].

(2) $$R_{tot}=\frac{TMP}{J\cdot \mu }$$

where *J* represents the permeate flux, *TMP* represents the transmembrane pressure and μ is the permeate viscosity, and the *R_tot_* is the filtration resistance. However, on the basic principle of Equation (2), *R_tot_* is a *TMP* and permeates flux *J* function. This is because the viscosity of the permeate is almost constant and equal to that of water.

## 4. Membrane Fouling Mitigation Strategies

An important area of study in membrane technology is to analyse fouling control mechanisms and develop simple methods to prevent or eliminate membrane fouling. Membrane surface properties significantly impact the fouling of membranes. Thus, the antifouling membrane design by properly tailoring the physicochemical properties can resolve this issue [76]. The techniques can usually be classified as passive and active. Passive antifouling strategies are created to avoid the early adsorption of foulants on the membrane surface without influencing the unique qualities of foulants. In contrast, active strategies tend to eliminate proliferative fouling by destroying the chemical properties and inactivating the cells. Therefore, comprehensive knowledge of various strategies and mechanisms for antifouling membrane surfaces is extremely important for surface modification.

### 4.1. Wastewater Pre-Treatment

Wastewater pre-treatment is an essential process that needs to be carried out in membrane filtration. This process is intended to eliminate organic and inorganic particles, which may damage the membrane structure. Furthermore, feed water is undergone pre-treatment to reduce the chances of membrane fouling. The key functions of pre-treatment techniques are to substantially reduce the amount of total suspended solids and different modes of fouling and scaling levels while maintaining membrane efficiency and life span [77]. In general, wastewater pre-treatment processes can be conducted by using conventional treatment processes and membrane-based pre-treatment processes.

#### 4.1.1. Conventional Treatment Process

A conventional pre-treatment involves several stages, including pH adjustment, coagulation, flocculation/sedimentation and filtration [78]. After pH adjustment, the coagulation process is commonly placed as the first pre-treatment step where coagulants/antiscalants such as alum are ordinarily mixed with the feed water. The addition of these coagulants/antiscalants can reduce the accumulation of matter on the surface of the membrane. However, it should be acknowledged that the concentration of the antiscalant should be carefully monitored as too high an amount of these chemicals may have negative effects on the membrane filtration cycle and the marine environment [79]. Consequently, flocculation or sedimentation is usually the primary unit after coagulation. At this stage, suspended particles are separated from the water. This happens due to the density difference between the suspended particles and water [80]. Finally, the remaining suspended particles are subsequently removed via filtration. Previous literature revealed that this non-conventional method efficiently rejects contaminants and successfully reduces SDI values and fouling issues in the RO membrane [81]. However, there are several drawbacks of the process, such as it requires large space, a high amount of chemicals and high cost. Therefore, to address this issue, the membrane-based method is introduced.

#### 4.1.2. Membrane-Based Method

Membrane-based methods such as MF and UF for feed water pre-treatment have been proven to achieve high efficiency in removing microorganisms, suspended matter, and colloids. The treatment also can achieve high removal of different contaminants and reduce SDI concentration and turbidity. Moreover, the cost-effectiveness of the membrane-based method is much higher compared to the conventional method. Ebrahim et al. [82] first showed that MF pre-treatment had shown promising alternatives in reducing fouling for membrane processes, as it has low permeate SDI with decrement percentage of biochemical oxygen demand (BOD) and chemical oxygen demand (COD). Coupling MF pre-treatment with chlorination unit has also been successfully investigated to mitigate biofouling [83]. Other than that, ceramic MF membrane for pre-treatment also has become a great interest among researchers. A porous ceramic MF membrane has proven to remove algae, microorganisms, and suspended solids during lake water treatment [84]. Besides that, hollow fibre membranes with capillary structures also received great attention as an alternative pre-treatment method in the membrane process to reduce fouling. In general, membrane fibres possess an internal diameter of 0.4 to 1.5 mm. The hollow-fibre membrane elements can be operated in either inside-out or outside-in flow patterns depending on the membrane manufacturer. An inside-out operating mode provides greater flow management and more consistent flow distribution than an out-in operation [85]. Due to the increased membrane per unit surface area, vacuum-driven membrane pre-treatment systems are typically more efficient than pressurised systems. Usually, membrane systems are driven by vacuum use up to 10 to 20% less space than membrane installations driven by pressure, assuming certain operating parameters [86]. Moreover, since a vacuum-driven membrane typically operates at lower trans-membrane pressure, their membrane fouling rate is lower, and they operate more stably during transient solid load conditions

Other than MF, UF pre-treatment is also considered a promising process to treat contaminated water, therefore mitigating the membrane fouling issue. This is mainly due to the small pore size of UF membranes which range from 0.01–0.1 μm, facilitating the removal of colloidal solids, aquatic colloids, microorganisms, organic and inorganic matter. Due to the effectiveness of the UF membrane, the development of UF pre-treatment for oily wastewater in RO significantly increased. For example, Salehi et al. [87] treated refinery oily wastewater using a hybrid UF/RO system. Particularly, the UF membrane system was developed as a pre-treatment for RO. As a result, the treated contaminated water by the UF pre-treatment process had an excellent quality to introduce to the RO process. Moreover, the final purified water at the RO outlet demonstrated up to 100% reductions of oil and grease with about more than 90% TOC, TDS, turbidity and BOD removal. Similarly, Arash et al. [88] reported that their γ-Al2O3 UF membranes exhibited good performance for oily wastewater pre-treatment. It can reduce the percentage of oil and grease content, TOC, BOD COD and turbidity by 84%, 67%, 63%, 73% and 79%, respectively.

### 4.2. Surface Modification

Surface membrane modification is one of the powerful techniques that can enhance membranes with desired properties. Compared to the common blending process, surface modification techniques provides a higher flexible means to enhance the surface properties while maintaining the base membrane bulk structure [89]. Surface modification has played a significant role in fabricating membranes with antifouling properties, as it increases the hydrophilicity of the membrane, reducing the possibility of fouling. Additionally, surface modification is preferable to modifying various membranes due to their economic cost. Two common surface modification methods that have been widely used to mitigate membrane fouling are surface coating and surface grafting. The surface coating modification method usually refers to coating a hydrophilic substance on the membrane surface. In contrast, surface grafting refers to the membrane surface modification by grafting polymer chains on the surface.

#### 4.2.1. Surface Coating

Surface coating is an easy and inexpensive process for surface functionalisation of the membrane and can be easily achieved in industrial and large-scale operations. Usually, the aim of fabricating a coating layer on the membrane surface is to provide long-term durability. The production of these membranes decreases expense and power consumption, as there is less surface heat loss between them [90]. However, some studies revealed that the coated layer on the surface of the membrane is brittle; thus, selecting the proper coating technique is essential [91]. In certain situations, treatment methods such as sulfonation or cross-linking on the surface of the membrane may be used to anchor the coated layer [92,93]. Many research studies have successfully improved water flux and antifoam rejection by hydrophilisation of membrane surface [29]. It was reported that coating hydrophilic materials on the PVDF UF membrane has achieved more than 90% flux recovery rate. Another study carried out by Zhao et al. [94] proved that the self-assembled coating of a hydrophilic layer onto polyvinylidene fluoride (PVDF) has increased antifouling properties of the fabricated membrane. In oily wastewater treatment applications, surface coating modification is excellent in preventing oil droplets from penetrating membrane pores to obtain a high water flux. Recently, titanium oxide (TiO_2_) has been coated into the alumina MF membrane to remove oil waste in water emulsion [95]. It was observed that the coating of TiO_2_ on MF membrane displayed higher flux compared to uncoated membrane, after 24 h separation, because of the high hydrophilicity of membrane-reduced membrane fouling. Besides that, Zhan et al. [96,97,98,99,100] developed a composite membrane using halloysite nanotubes (HNTs) with graphene oxide (GO) intercalation coated on porous poly(arylene ether nitrile) nanofiber to treat oil from wastewater. The composite membrane was then further enhanced with polydopamine (PDA) coating, which gave excellent oil separation with 99% rejection and 1130.56 L/m^2^h permeate flux. Similarly, Han et al. [21] reported 99% oil rejection by PDA coating. The high rejection of oil and permeate flux was attributed to the enhancement of membrane surface wettability, which reduced the attachment of small oil droplets.

#### 4.2.2. Surface Grafting

Surface grafting has been one of the surface modification techniques that creates covalent bonding interaction on the surface with new functional groups. Surface grafting can be performed via a chemical processor with high-energy radiation. However, it has been reported that surface grafting, besides the presence of additional functional groups, it could also alter pore structures. For instance, membrane pores may enlarge or shrink [101,102]. Therefore, various researchers have modified their novel membrane surface to treat oily wastewater over the last decades, such as how CA membranes have been grafted with polyacrylonitrile (PAN). The modification changed the surface morphology of the CA membrane, subsequently increasing the antifouling performance [99].

Other than polymers, hydrophilic nanoparticles are often integrated on the membrane’s surface through surface functionalisation [100]. In general terms, the hydrophilicity of the surface is enhanced by adding polar functional groups on the surface of the membrane. Subsequently, if the polar functional groups are immersed in water or oil, they turn inwards, thus reducing surface energy [101]. Membranes with superoleophobicity underwater have been studied. Once the grafted membrane is immersed in water, it can effectively reject oil and mitigate fouling to a certain extent. [100,102]. In recent years, surface grafting by ultraviolet (UV) irradiation of the membrane surface has also attracted more attention to increase the hydrophilicity of the membrane and mitigate the fouling issue during filtration [103,104]. For example, some researchers have applied UV irradiation grafting to introduce acrylic acid into the polymeric membrane, which greatly enhanced the hydrophilicity properties of the membrane [104].

Researchers have applied many modifier agents such as maleic anhydride, polyethylene glycol, and hydrophilic monomers [105,106]. For grafting a hyperbranched polyethylene glycol (HB-PEG), corona–air plasma was employed by Adib and Raisi [105]. They found that PEG increased the hydrophilicity of the membrane surface, which influenced the enhancement of the antifouling property without compromising oil rejection. The permeate flux from the resulting membrane increased from 91.8 to 99.5 L/m^2^h when the modified membrane was tested with 3000 ppm synthetic oily wastewater at 1.5 bar with an average droplet size of 570 nm. Furthermore, the FRR improved to 72% from the 56% of the unmodified PES ultrafiltration (UF) membrane, and the oil rejection was constant at 91.8%. Yuan et al. [106] grafted different molecular weights of propargyl PEG (pro-PEG) on to azide-functionalised polysulfone (PSF) membrane surfaces to treat oil emulsion. The functionalised membrane demonstrated high separation efficiency with 99.9% oil rejection. The reported flux using the grated membrane is 120 L/m^2^h. At the same time, it also achieved a 95% flux recovery flux, showing the good antifouling performance was attributed to the layer grafted on the membrane surface.

### 4.3. Optimisation of Membrane System Operating Conditions

In addition to membrane modification and pre-treatment of feed, operating conditions for oily wastewater treatment are also crucial for controlling fouling. Operational environmental factors such as hydrodynamic state, back pulse time, temperature, transmembrane pressure (TMP), and oily wastewater concentration can be controlled to prevent fouling formation [107]. In advance, the operating conditions for the membrane filtration system were optimised by deploying the full factorial design methodology. The different operating conditions were analysed concerning permeate flux, fouling resistance, and total organic compound (TOC) rejection [92]. As a result, the filtration module will have sufficient hydrodynamic conditions to reduce the fouling [108]. As aforementioned, the cross-flow configuration, for example, is reported to cause less fouling impact than the dead-end configuration [109]. Furthermore, usage of pulsed feed flows or other disruptions on the membrane surface, such as implemented continuous or pulsed electrical scopes, can effectively minimise membrane fouling [110].

On the contrary, for surface water treatment, it was observed that constant TMP operation resulted in less fouling at a certain operating temperature [32]. Oily wastewater that is high in concentration is highly prone to cause fouling. Pre-treatment such as flocculation or pre-filtering is helpful before filtration [111,112]. This operating condition should be optimised to achieve the best result for mitigating fouling in membrane separation technology. Mohammad et al. [113] first reported a study on oily wastewater effluent treatment using commercialised UF membrane with different operating conditions. Based on the research findings, the optimum operating conditions of UF membrane is at TMP more than 3 bar, the temperature of 30 °C and conducted under cross-flow configuration. Recently, an NF membrane was reported for fuel oil wastewater treatment under different temperature and oil concentration conditions to determine the optimum operating condition with the least fouling [114]. The optimum condition to obtain 100% removal purity and 65 L/m^2^h flux was established at 7 mg/L oil concentration and a temperature of 31 °C.

### 4.4. Membrane Cleaning Process

**Figure 3:**
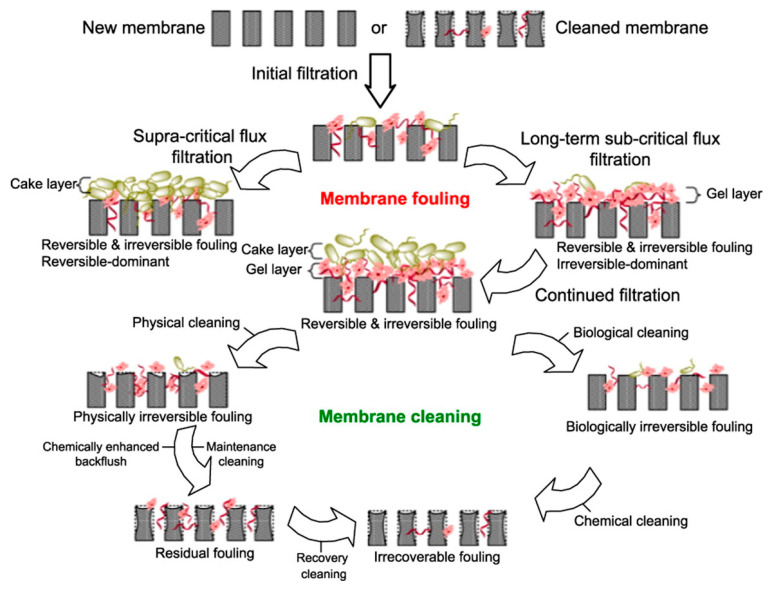
Illustration of membrane fouling types, mechanisms and the required cleaning techniques [124].

Membrane cleaning involves disrupting the foulant–membrane interactions. This process can be divided into physical and chemical cleaning [115]. Figure 3 shows the illustration of the required cleaning techniques for membrane fouling. For the case of physical cleaning, this can be conducted either by backflushing by controlling the stream rate and relaxation while preventing access of oil droplets into membrane pores. Besides backflushing, physical cleaning can also be carried out by using online ultrasonic [116], the inclusion of suspended particles and carriers [117], and mechanical cleaning, of which their comparison is depicted in Table 4.

Conversely, chemical cleaning is characterised by applying chemical agents, commonly from alkaline and acidic types, to mitigate irreversible membrane fouling. The function of the cleaning agent is to clean the foulant from the membrane surface and transfer it into the bulk solution [118]. Initially, Obeidani et al. [119] investigated the performance of different chemical agents used for MF membranes, including oxalic acid, caustic soda and sodium hypochlorite, to remove oil substances from contaminated seawater effluent. The results exhibited that acidic-based chemical agents have higher effectiveness than alkaline types. Conversely, Garmsiri et al. [120] reported that alkaline salts such as sodium hydroxide (NaOH) are also an efficient chemical cleaning process for MF membranes to treat oily wastewater.

Moreover, Zhu et al. [121] used NaOH solution to clean hollow-fibre MF membrane fouled by oil emulsion. The resulted membrane showed approximately 96% flux recovery after being used again. Surfactants and the chelating group can also be used as chemical agents. For example, cetyltrimethylammonium bromide (CTAB) was used as a cleaning agent for nanofiltration (NF) hollowfibre membranes [122]. After cleaning, it was found that the clean NF hollow-fibre membranes displayed a 100% flux recovery.

Nevertheless, the membrane cleaning process using conventional cleaning agents is time-consuming. Moreover, the process requires high operation costs. Therefore, the current alternative strategy that has been used is developing a photocatalytic membrane. Photocatalytic membrane offered an efficient separation performance in the oily wastewater treatment field and showed an excellent self-cleaning property under light irradiation without any additional cleaning agents. For example, Li et al. [123] fabricated a porous membrane based on the electrochemical formation of hierarchical TiO_2_ nanotubes on the surface of porous titanium for oily contaminated wastewater. They claimed that once the membrane was contaminated with organic molecules, the hydrophilicity of the membrane decreased. However, the wettability of the resultant membrane recovered by the induction of UV light, leading to increased recovery of permeate flux. Based on the study, the separation efficiency of several types of oil including gasoline, n-heptane and cyclohexane can achieve between 97.2% and 99.4% with 1357 L/m^2^h permeate flux.

**Table 4:** Different cleaning strategies [115,123].

Denomination	Description	Reference
Water washing	Manually carried out by shaker, where the fouled membrane is placed in a tank and shaken at a constant speed.	[119]
Ultrasonication	The membrane is placed in a tank and subjected to ultrasound washing, where the contact time and the power may vary as a function of fouling.	[119]
Sponge scrubbing	The membrane is cleaned using a sponge until clean	[119]
Photocatalytic cleaning	Photocatalytic materials are added to the membrane for self-cleaning under light irradiation purposes. The membrane is placed under the light before being reused for permeability test.	[125]

## 5. Future Outlook and Conclusions

Oily wastewater discharged by the industries needs to be treated before it can be fully discharged, as there are various forms of foulants present in real oily wastewater, including biofilms and organic and inorganic foulants [125]. Over the last decades, membrane separation technology has been regarded as one of the most effective treatments for oily wastewater. However, the main drawback of the membrane process is the fouling issue. Excellent progress in past studies has been demonstrated in designing various membranes with high antifouling properties. This review provides a brief view of factors that influence membrane fouling, including membrane properties and surfactants’ presence. A better understanding of fouling mechanisms as well as the mitigation strategies is further explained. Previous literature has proven the impact of opening pore size and surface roughness morphology upon this fouling mitigation property. Membranes with wide pore structures can result in high fouling as a result of pore-clogging.

Conversely, tailoring the physicochemical properties of the membrane will reduce the dynamic detachment of the surface of the membrane, while identifying the impact of a particular membrane structure upon these antifouling characteristics of the membranes, which is important. To further mitigate fouling, it is recommended to apply a pre-treatment system to oily wastewater before the filtration process or to combine various treatment methods to reduce membrane fouling [125]. To save space and cost, membrane-based pre-treatment such as MF and UF is preferable to obtain high removal of contaminants that significantly reduce any form of suspended particles or microorganisms from the contaminated water with low energy consumption. Further treatment can be performed to overcome the fouling problem during the separation of the oil–water process. Current mitigation strategies to deal with membrane fouling in the oily wastewater treatment field are modified by synthesised or commercial membranes via surface coating and surface grafting techniques. Most researchers use hydrophilic materials to prevent foulants from attaching to the modified membrane surface. However, the long-term stability of the modified layer of the membrane through coating and grafting is an important issue to be addressed. Optimising the operating parameters (i.e., back pulse time, temperature, transmembrane pressure) in the membrane system is another great alternative to prevent fouling formation with high separation efficiency. However, the conventional approach to optimise various parameters consumes considerable time and cost.

Furthermore, membrane cleaning strategies also possess excellent results in mitigating fouling. However, some chemical cleaning methods are considered hostile, as they can negatively affect the membrane. Although, a self-cleaning membrane by photocatalysis has been introduced as a green, economic and promising method to mitigate the fouling issue and retain the high permeate flux of membrane. Yet, the effect of light intensity and time for self-cleaning processes should be further studied.

## Abbreviations

Abbreviation/Nomenclature	Definition
BOD	biochemical oxygen demand
COD	chemical oxygen demand
CTAB	cetylrimethylammonium bromide
DAF	dissolved air floatation
GO	graphene oxide
HB-PEG	hyperbranch polyethylene gycol
HNTs	halloysite nanotube
MF	microfiltration
NaOH	sodium hydroxide
NF	nanofiltration
PEG	polyethylene glycol
PDA	polydopamine
PSF	polysulfone
RO	reverse osmosis
TiO_2_	titanium dioxide
TMP	transmembrane pressure
UF	ultrafiltration
UV	ultraviolet
